# Direct Aggression and the Balance between Status and Affection Goals in Adolescence

**DOI:** 10.1007/s10964-019-01166-0

**Published:** 2019-11-15

**Authors:** Jelle J. Sijtsema, Siegwart M. Lindenberg, Tiina J. Ojanen, Christina Salmivalli

**Affiliations:** 1 Tilburg University, Tilburg, The Netherlands; 2 University of Groningen, Groningen, The Netherlands; 3 University of South Florida, Tampa, FL USA; 4 Shandong Normal University, Jinan, China; 5 University of Turku, Turku, Finland

**Keywords:** Social goals, Direct aggression, Development, Status, Affection

## Abstract

Previous studies have shown that status goals motivate direct forms of interpersonal aggression. However, status goals have been studied mostly in isolation from affection goals. It is theorized that the means by which status and affection goals are satisfied change during adolescence, which can affect aggression. This is tested in a pooled sample of (pre)adolescents (*N* = 1536; 49% girls; ages 10–15), by examining associations between status goals and direct aggression and the moderating role of affection goals. As hypothesized, with increasing age, status goals were more strongly associated with direct aggression. Moreover, for older adolescents, status goals were only associated with aggression when affection goals were weak. These findings support the changing relationship between status goals and direct aggression during adolescence.

## Introduction

Direct interpersonal aggression, here defined as any overt behavior carried out intentionally to harm another person or damage their possessions, can be highly disruptive in social relationships and has sparked much research. These behaviors include verbal acts, such as shouting at someone or calling names, and physical acts, such as kicking, hitting, and pushing. Interestingly enough, direct aggression decreases in frequency during adolescence (e.g., Tremblay 2010). Prominent explanations of this decrease often point to changes in personality characteristics, such as impulsivity and sensation seeking (Wilson and Scarpa 2011), or focus on the influence of social circumstances on the display of direct aggression (Tremblay 2010). For example, the social learning theory of aggression (Bandura 1973) proposes that social goals may explain social influences on behavior. Goals can become more or less salient depending on either individual characteristics or social context. Hence, the same goal may be related to different behavior as a function of context (e.g., one where aggressive behaviors are rewarded compared to a context in which helping is rewarded) and individual characteristics (e.g., impulsive individuals may use aggression to satisfy goal attainment immediately). Yet, these views fail to explain the decrease of direct aggression during adolescence. Although these theories provide information on what goals are relevant or rewarding in which context, they say nothing about why and how the saliency and rewards change during adolescence, and hence they cannot explain the decrease of direct aggression. To explain the decrease of direct aggression during adolescence, it is thus needed to examine the interdependence of several social goals, i.e., the extent to which the satisfaction of one goal depends on the satisfaction of the other. In the current study, a goal-related theory was developed and tested that may explain the changing relation of social goals with direct aggression.

### Status and Affection Goals

There is increasing evidence that social goals play an important role in behavior (Lindenberg 2013) and social goals have attracted attention in youth research since the 1980s. Beginning with the pioneering work of Renshaw and Asher (1983), research focusing explicitly on goals has grown rapidly (e.g., Ojanen et al. 2005; Volk et al. 2012). In line with relationship studies (Clark and Mills 1979), most of this research focuses on two kinds of goals: communal goals, related to love and intimacy, referred to as *affection goals*, and agentic goals, related to power and dominance, referred to as *status goals* (see also Locke 2003). Although status and agency are not identical, in the context of the current study, they both refer to aiming for power and gaining respect from others. As currently measured, these trait-like goals reflect interpersonal motivations that come close to fundamental interpersonal needs (Lindenberg 2013), which are universal and important for human development (Anderson et al. 2015).

The goal framework is fruitful for the study of direct aggression, especially when focusing on contexts that are most frequent in our kind of society: contexts in which direct aggression is not related to survival goals but to social goals. During adolescence, school bullies have particularly strong status goals (Reijntjes et al. 2016; Sijtsema et al. 2009) and the pursuit of status goals has been linked to the use of direct aggression in adolescence over time (Caravita and Cillessen 2012; Ojanen and Findley-Van Nostrand 2014). Yet, the presence of status goals does not necessarily imply an absence of affection goals: status and affection goals are orthogonal rather than negatively correlated (Ojanen et al. 2005) and their simultaneous assessment may be particularly important in the study of direct aggression. Most individuals endorse both status and affection goals, to differing degrees (Steverink and Lindenberg 2006). For example, in several studies it has been argued that adolescents who want to achieve status often try to do so by not losing affection from significant others (Dijkstra et al. 2010; Sijtsema et al. 2010; Veenstra et al. 2010) and thus have to maintain a balance between satisfying both status and affection goals (cf. Tesser 1988).

Presently, existing theory and research on the link between social goals and direct aggression remains limited in three main ways. First, the relative salience and legitimacy of social goals that affect direct aggression are likely to change across development (Monahan et al. 2009) and thereby may affect goal-aggression links across adolescence. However, to date, goal-aggression links have not been examined from this perspective. Second, although theoretical views on goal-aggression links are prevalent, the main and interactive effects of particular social goals or motives on aggression remain poorly understood. Third, goals for social interaction are rarely measured directly. Hence, research on the association between status goals and direct aggression across adolescence would further benefit from consideration of various goal types, such as goals for status and affection, simultaneously. The significance of understanding potential age-related differences in the links between social goals and aggression lies (a) in its potential contribution for improving and designing developmentally appropriate interventions to counteract direct aggression, and (b) in the added insight into age-related changes in the social environment that impact direct aggression via the increasing overlap of social circles for achieving status and affection. In the discussion section, these points will be treated in some more detail.

### Direct Aggression, Status, and Affection

The general theoretical argument is the following. Status and affection are universal human goals (Lindenberg 2013), even though the strength of these goals may vary between individuals and contexts (cf. Crick and Dodge 1994). Direct aggression can be an effective means for achieving status. However, the use of aggression can also backfire and result in a loss of affection, especially when status and affection are derived from the same circle of interactions (e.g., the same peer group or classroom). This means that to understand the use of direct aggression, attention should be paid to the developmental changes in the context within which status and affection are realized. It is posited that, due to developmental changes (described below), the use of direct aggression for the achievement of status will increasingly jeopardize the realization of affection. Thus, during adolescence, direct aggression for the purpose of status will become more costly (in terms of losing affection and decreasing legitimacy as a means for achieving status) and therefore diminish. Please note that the current focus is specifically on direct forms of aggression, to the exclusion of indirect (or covert, relational) forms of aggression. This is important for two reasons. First, indirect aggression is more likely to be used to achieve both status and affection goals (Heilbron and Prinstein 2008). Second, direct and indirect aggression show different developmental profiles across adolescence: direct aggression becomes less frequent whereas indirect aggression becomes more adaptive and hence more frequent during adolescence (e.g., Cillessen and Mayeux 2004).

### The Changing Link between Status Goals and Aggression

Concerning the relationship of direct aggression to status and affection goals, it can be argued that during preadolescence, youths may realize affection and status within the same group of peers to some extent. However, they often avoid loss of peer affection by being aggressive towards those who were rejected by significant others (Veenstra et al. 2013). In addition, at this age, parents are a focal provider of love and warmth and may thus be an effective alternative source of affection when affection from peers is lost due to the pursuit of status. There is some indirect support for this proposition. In young adolescents, parental warmth and rejection were much more important for behavioral and emotional adjustment than peer acceptance and rejection (Sentse et al. 2010). Compared to the period of adolescence, such parental effects are likely to be stronger in preadolescence, given the more central role of parents (Nangle et al. 2003). Also in other research it was shown that children are able to use direct aggression in the peer context for the pursuit of status without much loss of affection (Huitsing and Monks 2018).

Previous work on the link between aggression and social status shows indeed that direct aggression in preadolescence is associated with high popularity, but also with low acceptance (e.g., Cillessen and Mayeux 2004; so-called ‘coercive controllers’, Hawley 2003). Other work that examined bullying in preadolescents, showed that bullying was associated with high perceived popularity, and that most bullies were neither particularly high nor low on social acceptance (Reijntjes et al. 2013). Together, these findings suggest that in preadolescence, among peers, status goals can be realized via direct aggression, but that it is more difficult to simultaneously satisfy affection goals in these circles of interactions.

Later in adolescence, parents are still important for affection, but peers are likely to take on an important role for the realization of both status and affection goals and it becomes difficult to divide peers into circles for affection and circles for domination. As youths mature physically, influences of mating become important, including competitive contexts that drive up the salience of status (de Bruyn et al. 2012). Adolescents want to belong to the in-crowd but, once accepted, they also want to rise in status within the in-crowd, and do whatever is necessary to achieve this (see Veenstra et al. 2010). This means that the use of direct aggression for the realization of status becomes more costly in terms of losing affection (e.g., Dijkstra et al. 2009) and that adolescents will, by and large, be more reluctant to use direct aggression to achieve status. If they pursue status via direct aggression nonetheless, they are likely to have relatively weak affection goals.

## The Current Study

Previous work suggests that the social circles in which adolescents fulfill status and affection needs become more integrated with age. This integration has consequences for the use of direct aggression. In preadolescence, direct aggression may be used within the peer context to achieve status, without running the risk of not receiving affection. This is because, in preadolescence, parents or caregivers still take up a central role in the provision of affection. In adolescence, the peer group plays a more dominant role in fulfilling social needs. Using direct aggression to gain status thus becomes more risky, as status and affection are derived from the same social group, namely peers. In this study, it is therefore argued that status goals are associated with direct aggressive behavior in adolescence, but that this association is contingent upon affection goals and age. The first hypothesis is that in younger adolescents, status goals are positively associated with direct aggression, irrespective of the level of affection goals. The second hypothesis is that for older adolescents, status goals are positively associated with direct aggression only at low levels of affection goals. Because previous work indicated significant differences between boys and girls in both levels of status and affection goals and direct aggression (Sijtsema et al. 2009), the role of sex is also explored.

## Methods

### Participants and Procedure

#### Sample 1

In this cross-sectional study, participants were 589 preadolescent fifth- and sixth-grade children (52.6% boys), 11–12 and 12–13 years of age, in 26 school classes from 8 different schools from a medium-sized town in southwest Finland (Salimivalli et al. 2005). Class sizes varied from 15 to 32 students.

#### Sample 2

In this cross-sectional study, participants were 255 preadolescent fourth graders (10–11-year olds; 49.2% boys) and 277 adolescent eighth graders (14–15-year olds; 44.9% boys) in 25 school classes from a small-sized town in the southwest of Finland (Sijtsema et al. 2009). Class sizes varied from 15 to 28 students.

#### Sample 3

Participants were 384 students (53% boys) in 25 school classes in two local middle schools from a medium-size town in Southeast Finland (see also Ojanen and Findley-Van Nostrand 2014). Class sizes varied from 9 to 20 students. Data from participants at wave 3 (*n* = 310) were used in the current study, when participants were 14–15 years old.

All samples were representative of most Finnish schools, which represent all social classes, from working class to lower- and upper-middle socioeconomic classes, with no large between-school socioeconomic differences. The students in all samples were predominantly of Finnish origin (96–98%). Participants filled out a questionnaire concerning aggression, goals, and status. In addition to self-reports, peer nominations were also collected. Consent forms were sent to parents, who were asked to return the form if they did not want their child to participate. For Sample 1, only 1.8% of the students in the participating classes (*n* = 11) did not receive parental permission to participate. In Sample 2, 31 (0.6 %) students were excluded from the study due to missing data or not receiving parental consent. For Sample 3, consent return rates were as follows: T1 = 85%, T2 = 72%, and T3 = 70%. Because new participants were allowed to enter while some dropped out from the project over time, participation rates varied across the measurements: T1: *N* = 384, T2: *N* = 315, T3: *N* = 310. Attrition analysis indicated no mean-level differences in status goal vector scores, *t*(384) = 0.56, *p* = 0.58, or affection goal vector scores, *t*(384) = 0.30, *p* = 0.77, between participants who remained in the project throughout the study and those who dropped out.

In sum, the final pooled sample consisted of 1536 participants (49% girls) with an average age of 12.56 (SD = 1.79). One participant did not report its sex. Analyses were performed on 1368 participants, who had full information on all study variables. Independent samples *t*-tests showed that participants with full information did not differ from those with missing data on status goals, affection goals, and direct aggression. Participants with missing data were somewhat older compared to those with full information (*t*(1534) = −13.77, *p<* 0.001), which is due to the fact that most participants with missing data came from Sample 3.

In all samples, participants were fully informed about the nature of the study, that participation was voluntary, and that they could withdraw at any moment. Data collection took place in classrooms during school hours, supervised by one or two researchers. These research procedures are in line with the ethical standards and guidelines in Finland, following the Declaration of Helsinki (1964) on ethical principles.

### Instruments

#### Direct aggression

Direct aggression was assessed via cross-sex peer nominations in Sample 1 and with same-sex peer nominations in Sample 2, with which participants checked off the names of their classmates who behaved in the ways described in the items. In Samples 1 and 2 there were six items pertaining to direct aggression (e.g., “when teased fights back”; “uses physical force to dominate”; *α* = 0.93 in Sample 1 and *α* = 0.77 in Sample 2) (Dodge and Coie 1987). The number of nominations a child received for each item was divided by the number of classmates who were present and doing the evaluation. Scale scores were the average scores computed for the six items.

In Sample 3, participants were asked to nominate up to 10 classmates (cross-sex) from their homeroom who fit each item (see Ojanen and Findley-Van Nostrand 2014). Being able to nominate 10 peers was considered non-restricting relative to class sizes. Two items were used to measure aggression (“fights with others” and “pushes, kicks, or punches others”; *α* = 0.93). Nominations for each item were summed and standardized by the number of participants in each class conducting the evaluation.

To aggregate these slightly different peer assessments of direct aggression, scores were standardized to a mean of zero and a standard deviation of one within each sample. Subsequently, these standardized scores were merged into one pooled dataset and used as the dependent variables in the analyses.

The use of peer nominations offers several advantages over other informant reports, such as self-, parent-, or teacher-reports, and may produce a more valid assessment (Cillessen and Marks 2017). Peer nominations constitute observations from (almost) all group members and thus are not limited to one viewpoint. Moreover, peers are often better at detecting low frequency behaviors, such as direct aggression, and peer nominations have high ecological validity as they often reflect how individuals behave across the day and in multiple contexts (in- and outside school, both in class and during breaks).

#### Status and affection goals

Social goals in the peer context were assessed with the Interpersonal Goals Inventory for Children, IGI-C (Ojanen et al. 2005). Under the frame “When with my peers, it is important to me that…,” participants were asked to rate the subjective importance of 33 interpersonal outcomes on a 4-point Likert scale, ranging from 0 (*not important to me at all*) to 3 (*very important to me*). This measure was developed based on the interpersonal circumplex model and respective measures in adults (Dryer and Horowitz 1997). The IGI-C has evidenced criterion validity in terms of various self-, peer-, and teacher-reported constructs across studies and cultures (Caravita and Cillessen 2012; Salmivalli et al. 2005; Thomaes et al. 2008) and includes eight subscales assessing goals in varying degrees of agency (status and power) and communion (closeness and affiliation) (Ojanen et al. 2005). The subscales are Agentic (e.g., “Others respect and admire you”), Submissive (e.g., “Others do not get angry with you”), Communal (e.g., “You feel close to one another”), Separate (e.g., “You don’t let your peers know how you feel”), Communal and Agentic (e.g., “Others listen to what you have to say”), Separate and Agentic (e.g., “You get to decide what to play”), Communal and Submissive (e.g., “The others accept you”), and Separate and Submissive (e.g., “You don’t do anything foolish”). In all three samples, all scales were internally consistent with alphas ranging from 0.60 to 0.77, with the exception of the scale Communal and Submissive goals which had somewhat lower internal consistency (α = 0.55 and 0.57 in Sample 2 and 3, respectively).

Following existing literature (e.g., Ojanen et al. 2005; Thomaes et al. 2008), information represented in the eight goal scales was summarized into overarching status and affection goal vector scores in the circumplex space (Locke 2003):

$${\mathrm{Status}}\,{\mathrm{Goals}} = Agentic \,-\, Submissive \,+\, \left[ {0.707\,\times\,\left(Communal\,and\,Agentic + Separate\,and\,Agentic-\,Communal\,and\,Submissive \, - Separate\,and\,Submissive\right)} \right]$$

$${\mathrm{Affection}}\,{\mathrm{Goals}} = Communal - Separate \,+ \left[ {0.707\times\left( Communal\,and\,Agentic + Communal\,and\,Submissive \,- \, Separate\,and\,Agentic - Separate\,and\,Submissive \right)} \right]$$

These scores were used to assess status and affection goals respectively. Scores on the four intermediate scales (Communal and Agentic, Communal and Submissive, Separate and Agentic, and Separate and Submissive) were multiplied by 0.707 because this is the cosine of a 45° angle (the angle of those scales, relative to the status and affection goal vectors).

### Data Analysis

First, data on social goals and aggression were standardized within each sample and then pooled. Next, because the distributions of direct aggression were skewed, bootstrapped correlation analyses were conducted, creating 1000 random samples to ensure the robustness of the results. This procedure yielded more reliable coefficients and 95% confidence intervals. Second, bootstrapped linear regression analyses were performed to test the hypotheses regarding the interaction between status and affection goals and age. Significant interactions were plotted using simple slope analysis (Aiken and West 1991). To reduce problems with multicollinearity and to ensure that the values plotted in the figures are accurate representations of the data, independent variables were centered (Frazier et al. 2004). Moreover, sex was controlled for in all analyses and interactions with sex were explored. All analyses were performed in IBM SPSS 24.0 and hypotheses were tested two-sidedly using a *p*-value of <0.05 to indicate significance. To compare the relative effect size of the main and interaction effects, semi-partial correlations squared were calculated as an index of explained variance.

## Results

In Table 1, means, standard deviations, and correlations of all study variables are presented for the separate samples and the pooled sample. Correlations in the pooled sample indicated that stronger status goals were associated with more peer-reported direct aggression, whereas stronger affection goals were associated with less peer-reported direct aggression. Age was positively associated with status and affection goals, suggesting social goals are more pronounced in older adolescents, compared to younger adolescents. Correlations in the separate samples pointed all in the same direction.

**Table 1:** Correlations, means, and standard deviations of the pooled and separate samples

Pooled sample	1	2	3	M	SD
1. Status goals	–			0.00	*1.00*
2. Affection goals	0.01	–		−0.00	*1.00*
3. Direct aggression	0.13**	−0.14**	–	0.00	*1.00*
4. Age (in years)	0.17**	0.12**	0.01	12.4	*1.75*

				Age 10.5 (below diagonal)		Age 12.5 (above diagonal)	
Sample 1	1	2	3	M	SD	M	SD
1. Status goals	–	−0.14*	0.14*	−1.53	*1.29*	−1.46	*1.26*
2. Affection goals	0.09	–	−0.10	1.96	*1.45*	2.00	*1.56*
3. Direct aggression	0.12	−0.02	–	0.20	*0.28*	0.25	*0.33*

				Age 10.5 (below diagonal)		Age 14.5 (above diagonal)	
Sample 2	1	2	3	M	*SD*	M	SD
1. Status goals	–	−0.15**	0.16**	−1.65	*1.16*	−0.75	*1.13*
2. Affection goals	0.07	–	−0.10	2.00	*1.23*	2.68	*1.48*
3. Direct aggression	0.07	−0.15*	–	0.11	*0.12*	0.10	*0.14*

				Age 14–15	
Sample 3	1	2	3	M	SD
1. Status goals	–			−1.24	*1.90*
2. Affection goals	0.07	–		3.07	*2.06*
3. Direct aggression	0.07	−0.15**	–	0.06	*0.17*
4. Age (in years)	0.08	0.16**	−0.05	14.47	*0.50*

*p< 0.05, **p< 0.01

**Fig. 1:**
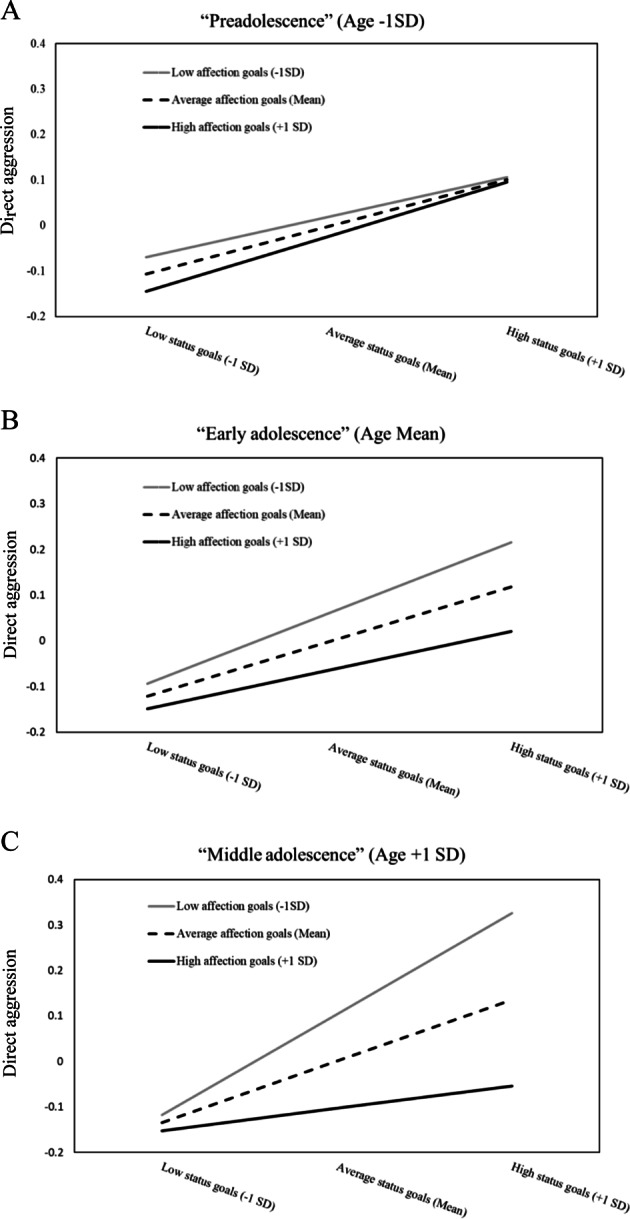
**a**–**c**. Associations between status goals and direct peer-reported aggression at low (−1 SD), average (mean), and high (+1 SD) levels of affection goals, plotted at different ages in A (‘Preadolescence’, −1 SD), B (‘Early adolescence’, Average), and C (‘Middle adolescence’, +1 SD)

Next, it was tested to what extent the changing link between status goals and direct aggression was contingent upon affection goals and age. In Table 2, main and interaction effects are presented of social goals and age on direct aggression, while controlling for sex. The last step included the three-way interaction between status goals, affection goals, and age. In line with the hypotheses, status goals were associated with peer-reported direct aggression, but only when affection goals were weak. However, this interaction was contingent upon age (*b* = −0.05, *SE* = 0.02, *p=* 0.01, 95% CI = −0.09 to −0.02, sr^2^ = 0.003). Simple slope analyses showed that status goals were positively associated with direct aggression, irrespective of the level of affection goals in ‘preadolescents’ (see Fig. 1a) and ‘young adolescents’ aged around the mean (see Fig. 1b). By contrast and in line with the hypothesis, in ‘middle adolescence’ (the oldest age group), status goals were positively associated with direct aggression only at low levels of affection goals (Fig. 1c; *b* = 0.22, SE = 0.05, *p* < 0.001, 95% CI = 0.12 to 0.32). Tests for the Johnson–Neyman significance region suggest that the interaction between status and affection goals became significant from around 14 years of age, which comprised 35.7% of the sample.

**Table 2:** Main and interaction effects of age, status goals, and affection goals on direct peer-reported aggression

	B	SE	95% CI	sr^2^
Main effects				
Constant	−0.17	0.04**	−0.23, 0.09	
Sex (1 = boy; 0 = girl)	0.32	0.06**	0.20, 0.44	0.02
Age (in years)	−0.00	*0.03*	−0.05, 0.05	0.00
Status goals	0.12	0.02**	0.07, 0.17	0.01
Affection goals	−0.08	0.03*	−0.14, −0.02	0.01
*R*^2^	5.8%			
Two-way interactions				
Constant	−0.17	0.04**	−0.23, 0.09	
Sex (1 = boy; 2 = girl)	0.32	0.06**	0.19, 0.44	0.02
Age (in years)	0.00	*0.03*	−0.05, 0.05	0.00
Status goals	0.12	0.02**	0.07, 0.17	0.01
Affection goals	−0.08	0.03*	−0.14, −0.02	0.01
Age × Status goals	0.02	*0.03*	−0.03, 0.07	0.00
Age × Affection goals	−0.04	*0.03*†	−0.09, 0.01	0.00
Status × Affection goals	−0.04	*0.02*†	−0.08, 0.00	0.00
*R*^2^	6.2%			
Three-way interactions				
Constant	−0.16	0.04**	−0.22, −0.09	
Sex (1 = boy; 2 = girl)	0.32	0.06**	0.20, 0.44	0.02
Age (in years)	0.00	*0.03*	−0.05, 0.06	0.00
Status goals	0.12	0.03**	0.07, 0.17	0.01
Affection goals	−0.07	0.03*	−0.13, −0.01	0.00
Age × Status goals	0.02	*0.03*	−0.03, 0.07	0.00
Age × Affection goals	−0.04	*0.03*^†^	−0.09, 0.01	0.00
Status × Affection goals	−0.04	*0.02*^†^	−0.08, −0.00	0.00
Age × Status × Affection goals	−0.05	0.02*	−0.09, −0.02	0.00
*R*^2^	6.5%			

^†^*p<* 0.10, *p< 0.05, **p< 0.01

Estimates are based upon bootstrapped regression models (*n* = 1000)

Sex differences in the associations between status goals, affection goals, and peer-reported direct aggression were also explored (not reported in the tables). Testing these interactions yielded one significant moderation. The positive association between status goals and peer-reported direct aggression was significantly stronger for boys compared to girls (*b=* 0.16, *p<* 0.01, 95% CI = 0.04 to 0.27). Sex did not moderate associations between status goals, affection goals, and age in explaining direct aggression.

Furthermore, sensitivity analyses were performed to check the robustness of the current findings. To this end, regression analyses were conducted on a randomly selected half of the pooled sample. This procedure was repeated 10 times. The three-way interaction between status goals, affection goals, and age was replicated in 5 out of 10 iterations and the two-way interaction between status goals and affection goals was replicated in 7 out of 10 iterations. It should be noted that statistical power to detect three-way interactions is considerably lower in these analyses because the analyses are performed on half of the total sample.

To provide full disclosure of the analyses that were performed, it was also explored to what extent the association between status goals, affection goals, and age could explain peer-reported indirect aggression. Peer-reported indirect aggression was collected in a similar way as direct aggression, but was only available in Sample 2 and 3 (*n* = 782). Status goals were positively associated with indirect aggression, but this association was not moderated by affection goals and age. Affection goals were negatively associated with indirect aggression and this negative association became stronger with age.

## Discussion

Although many studies have reported a decrease of direct aggression in adolescence, the reasons for this decrease have remained somewhat of a puzzle. To address this issue, it was examined to what extent the decrease in direct aggression during adolescence can be explained by focusing on the interdependence of status and affection goals. Adolescents have both of these social goals and their realization is likely to have a profound impact on the interaction among peers. On the one hand, direct aggression can be an effective way to realize status. At the same time, direct aggression also lowers affection from the targets of aggression as well as from other peers. As the social circles of interaction within which status and affection are realized become more integrated during adolescence due to developmental changes, i.e., both affection and status are to a large extent provided by the peer group, the use of direct aggression for realizing status becomes increasingly costly in terms of losing affection. Thus, by middle adolescence, when the realization of status and affection goals have become dependent on the same circle of peers, socially determined direct aggression should be explained by the relative strength of status and affection goals: the combination of strong status and relatively weak affection goals.

A preliminary test was provided of these age-specific hypotheses using unique secondary data that contained, next to direct aggression, measures of both status and affection goals. In line with the hypotheses, it was shown that status goals are associated with direct aggression throughout adolescence. This finding echoes previous empirical research, showing that at this age status can be pursued via direct forms of aggression (Caravita and Cillessen 2012; Ojanen and Findley-Van Nostrand 2014). It was also found that, as hypothesized, in preadolescence and early adolescence, affection goals do not affect the association between status goals and direct aggression. This is in line with earlier work showing that at young ages, children seem to be able to use direct aggression without much concern for the loss of affection (Huitsing and Monks 2018). Conversely, the current findings corroborated the hypothesis that for older adolescents, status goals are more strongly associated with direct aggression, but only when youths have lower levels of affection goals. Because having weak affection goals is relatively rare, direct aggression decreases for older adolescents compared to preadolescence and early adolescence.

### Implications

The larger significance of these findings may lie in what they suggest about the age-related development of direct aggression and with regard to possible interventions to reduce the likelihood of direct aggression. Based on the current research, it seems worth-while to pay attention to the overlap of social circles in which status and affection are realized. This overlap may differ for different contexts in and out of school, in an out of work, et cetera. To the degree that they overlap, direct aggression is less likely caused by the social context. The overlap increases less and less with age for the achievement of status because of the stronger overlap of status and affection social circles. This overlap means that direct aggression will not lead to the desired result; that it is not considered acceptable; and that there are more varied alternatives to achieve status without losing affection. If true, direct aggression would with age become increasingly a matter of personality characteristics rather than social context.

An important caveat is a possibly temporary contrasting effect of an *increased* link between status and aggression in mid-adolescence because in this developmental period directly aggressive youths represent a challenge to adult roles and values (Pellegrini and Long 2002). Thus, one might speculate that this in-group-out-group effect of youths versus adults makes the use of aggression for the achievement of status less costly, creating what Hawley et al. (2008) called the “the peer regard–aggression paradox”, an effect that was also found by others (Dijkstra et al. 2008). However, in support of the current findings, this increased acceptability of aggression for the achievement of status may be rather temporary and could wear off as peer and romantic relationships become increasingly important (see also Pellegrini and Long 2002).

With regard to interventions, the current research encourages the development of programs in schools that explicitly deal with the realization of status and affection. For example, this relationship could be explicitly discussed in classrooms, and it could be emphasized that the use of direct aggression for the achievement of status is not only counterproductive (in terms of loss of affection) but also a display of behavior that is done by young children rather than by more mature people. In this way, the very use of direct aggression may become counterproductive in terms of status. In addition, schools could target youths who have strong status and weak affection goals. Specifically for them, alternative ways to achieve status may be devised (see for example Ellis et al. 2016). Conversely, schools may try to increase the salience of affection goals. For example, self-disclosure, known to facilitate affective relationships, could be trained (Tokic and Pecnik 2010), along with training in empathy (Zaki 2019).

Future research may expand on the current findings in a number of ways. For example, it may focus explicitly on changes in the ways affection is realized, and trace systematic differences in various social contexts with regard to the changes in overlap of social circles in which status and affection are realized. Importantly, intergroup relations might be particularly interesting. For example, Coleman (1961) showed that in the United States, interscholastic competition in non-violent sports led to an increased compatibility of status (based on skills for winning from the out-group) and affection within the in-group. Quite generally, the availability of multiple ways to achieve status and their relationship to the achievement of affection could become a focus for future research.

### Limitations and Strengths

The findings should be interpreted against the backdrop of several limitations. First, peer-reported aggression was assessed in slightly different ways in the three samples. In Samples 1 and 3, same- and cross-sex peer nominations were used to assess direct aggression, whereas in Sample 2 peer nominations were only administered for same-sex peers. In addition, compared to Samples 1 and 2, in Sample 3 a shorter instrument was used to assess direct aggression with slightly different items. Yet, all items clearly assessed physical, observable forms of aggression or the threat thereof. Moreover, merging different samples to study goal-aggression associations and mapping age-related differences in these links means that cohort effects cannot be excluded and that changes within and between individuals cannot be studied. Thus, future research would benefit from assessing longitudinal relationships between social goals and direct aggression in a longitudinal design that allows for studying these developments during adolescence within the same set of individuals.

Next, the subscale Communal and Submissive goals that was used to calculate the status and affection goal vector scores, showed low internal consistency. It could be that this subscale reflects a somewhat broader construct that cannot be assessed easily with only four items. It is thus important to replicate the current findings with a more optimal assessment of social goals.

Although the three samples included youths from diverse socioeconomic backgrounds, all studies were conducted in Finland, which limits the generalizability of the current findings to other cultures and ethnicities. Moreover, Finland is a country that invests much in reducing aggression in school (Persson et al. 2018), which may influence how aggression is viewed by youths and thus how it is related to social goals.

However, one message of this study is that attention should be paid to the overlap of circles for achieving status and affection. These overlaps do not just differ by age (this study), but very likely also by culture and other social circumstances. For example, clique formation in classrooms may allow status achievement via aggression towards other cliques, while realizing affection inside one’s own clique (Pattiselanno et al. 2016). Thus, clique formation (possibly based on heterogeneity in classrooms) may keep status and affection circles further separated for a longer time in the developmental process. In addition, it cannot be excluded that in certain communities, rates of direct aggression may be higher and are driven by goals and motivations related to retaliation and survival (e.g., in low-income urban areas, see Aceves et al. 2010). In these contexts, the link between status goals and direct aggression may be less contingent upon having weak affection goals, but could reflect individuals’ willingness to fight back and stand their ground when teachers fail to intervene adequately (see also Veenstra et al. 2014).

Finally, although the current study captured a substantial part of adolescence, there was no information on (early) childhood and late adolescence (i.e., age 16–18 years). If the theoretical notions and empirical findings hold, it would be expected that in childhood parents play an important role in both shaping and satisfying social goals. At that age, both status and affection goals may be difficult to assess, may not yet be clearly present, and may not be related to direct aggression. In late adolescence, it can be expected that the interaction between status and affection goals is more pronounced when it comes to explaining direct aggression. Future research is needed to extend the current research by assessing social goals in a larger age range in a longitudinal design.

Notwithstanding these limitations, a methodological strength of this project was that status and affection goals were assessed directly and via the same instrument across multiple studies. Measuring social goals or motives for social interaction directly is still rare and using the same instrument across studies may excuse a number of minor methodological differences between them. Moreover, a coherent goal-related framework was provided for tracing social influences on the use of direct aggression, linked to an age-increasing integration of social circles within which status and affection goals are being realized. Finally, hypotheses were empirically tested about age effects concerning social determinants of direct aggression. It was shown that the association between status goals and direct aggression changes across adolescence as a function of the importance of affection goals and the concomitant cost of achieving status by direct aggression.

## Conclusion

To explain the decrease in frequency of direct aggression, a goal-related theory was tested and developed that could explain the changing relation of social goals with direct aggression. It was found that status goals are related to increased direct aggression also later in adolescence, but only when affection goals are weak. Yet, it is important to note that the effects were small and other factors may account for much of the variation in direct aggression. That is, direct aggression may be more directly explained by negative interactions between neuropsychological deficits and adverse environments, possibly linked to a life-course persistent trajectory of antisocial behavior and problem behaviors in general (Moffitt 2018). The current findings may have implications for policies and interventions that target direct aggression. For one, it is important to realize that status goals by themselves are not necessarily related to more direct aggression later in adolescence. However, coupled with low affection goals, youths may lack the motivation to inhibit the use of direct aggression. Interventions may thus want to identify such youths and strengthen the salience of affection goals to counteract the negative effect of status goals.
